# Are health care assistants part of the long-term solution to the nursing workforce deficit in Kenya?

**DOI:** 10.1186/s12960-020-00523-6

**Published:** 2020-10-20

**Authors:** Louise Fitzgerald, David Gathara, Jacob McKnight, Jacinta Nzinga, Mike English

**Affiliations:** 1 De Monfort University, Leicester, UK; 2 KEMRI-Wellcome Trust Research Programme, Nairobi, Kenya; 3 Nuffield Dept. of Medicine, University of Oxford, Oxford, UK; 4 KEMRI-Wellcome Trust, Nairobi, Kenya; 5 NDM, University of Oxford, Oxford, UK; 6 Health Services and Research Group, KEMRI-Wellcome Trust, Kenya Medical Research Institute/Wellcome Trust Research Programme, PO Box 43640, Nairobi, 00100 Kenya

**Keywords:** Health care assistants, Nursing practice, Task shifting, Skill mix, Workforce deficits

## Abstract

This commentary article addresses a critical issue facing Kenya and other Low- and Middle-Income Countries (LMIC): how to remedy deficits in hospitals’ nursing workforce. Would employing health care assistants (HCAs) provide a partial solution? This article first gives a brief introduction to the Kenyan context and then explores the development of workforce roles to support nurses in Europe to highlight the diversity of these roles. Our introduction pinpoints that pressures to maintain or restrict costs have led to a wide variety of formal and informal task shifting from nurses to some form of HCA in the EU with differences noted in issues of appropriate skill mix, training, accountability, and regulation of HCA. Next, we draw from a suite of recent studies in hospitals in Kenya which illustrate nursing practices in a highly pressurized context. The studies took place in neo-natal wards in Kenyan hospitals between 2015 and 2018 and in a system with no legal or regulatory basis for task shifting to HCAs. We proffer data on why and how nurses informally delegate tasks to others in the public sector and the decision-making processes of nurses and frame this evidence in the specific contextual conditions. In the conclusion, the paper aims to deepen the debates on developing human resources for health. We argue that despite the urgent pressures to address glaring workforce deficits in Kenya and other LMIC, caution needs to be exercised in implementing changes to nursing practices through the introduction of HCAs. The evidence from EU suggests that the rapid growth in the employment of HCA has created crucial issues which need addressing. These include clearly defining the scope of practice and developing the appropriate skill mix between nurses and HCAs to match the specific health system context. Moreover, we suggest efforts to develop and implement such roles should be carefully designed and rigorously evaluated to inform continuing policy development.

## Introduction

Kenya and other Low- and Middle-Income Countries (LMICs) face many dilemmas in developing their health care systems. Some of these are unique to each country, but others are common across High-Income Countries (HICs) and LMICs. This commentary article focuses attention on a contemporary issue—whether employing health care assistants (HCAs) would be helpful when hospitals have nursing deficits. To raise debate, we provide reference data on the growth in employment and diverse nature of HCAs in Europe and draw on a detailed body of work conducted in Kenyan hospitals.

Throughout, we use the umbrella term HCA for those who provide direct personal care and assistance with activities of daily living to patients and residents in a variety of health care settings such as hospitals, clinics, and residential nursing care facilities. They generally work in implementation of established care plans and practices, and under the direct supervision of medical, nursing, or other health professionals or associate professionals [1].

In Kenya, nurse aides existed in the past, but this cadre was abandoned over concerns that some were misrepresenting themselves as fully qualified staff and with an increasing drive towards fully professionalising nursing, including the introduction of degree level training and efforts to eliminate training at certificate level. Currently, there is no legal or recognised regulatory provision for HCA in Kenya. We use data from recent studies of nursing practice undertaken in neo-natal wards [2–5] to illustrate the severe pressures in Kenyan county hospitals (typically 80–400 bed general hospitals) where nurses carry extreme workloads. A direct result is their delegation of tasks to largely unqualified ‘helpers’.

Extreme workloads in the Kenyan public-sector result from nurse-to-population ratios that are very low, ranging from 0.008 to 1.2 nurses per 1000. The comparable figures are 5.51, 6.54, 7.86, 10.46, and 16.9 nurses per 1000 population in Spain, Italy, UK, France, and Denmark, respectively [6]. In Kenyan public hospitals, a single nurse may often be responsible for more than 20 patients, including on neo-natal wards where UK ratios would be between 1:1 and 1:4 depending on patient acuity. Paradoxically, Kenya has many unemployed nurses, but there is little evidence, suggesting that significant new financing will be made available to dramatically increase public-sector recruitment despite evidence that lower nurse ratios negatively impact the quality of care [2, 7]. The World Health Organization (WHO) suggests task shifting and sharing as one means to ameliorate HRH shortages while potentially improving access and maintaining or improving quality [8]. Re-introduction of a form of HCA in Kenya might therefore be considered; however, designing task-shifting solutions should take account of context and could usefully be informed by experience in other settings.

The dire need for expanded human resources in healthcare is not specific to Kenya. Globally, healthcare systems face pressures to sustain quality health care against rising costs, innovation, and increasing demand by providing appropriate numbers of health workers with the right skill mix to offer safe, quality care. Of the 43.5 million health workers in the world, it is estimated that 20.7 million (almost 50%) are nurses and midwives. Yet, 50% of WHO Member States report having less than 3 nursing and midwifery personnel per 1000 population [9]. Although nurses are critical to the provision of quality healthcare and their numbers directly impact patient outcomes, many countries are struggling to address nursing workforce gaps [7, 10].

The result has been an international trend to employ HCAs to support nurses. As a reference framework, we draw on data from the European Union (EU) and especially the UK, where employing HCAs is widespread and the role has been researched. The Kenyan and UK health systems have strong historical links and, as stated, Kenya introduced policies to increasingly professionalise nursing, such that over 80% of nurse trainees are now at diploma level and degree level training is increasingly common. HCA role development in the UK system is deemed especially relevant as an unregulated HCA role has emerged in its public system. Nevertheless, we acknowledge the considerable differences between the UK and Kenya and so position UK experience within wider evidence on and roles and regulation of HCA in the EU to demonstrate the considerable diversity in nursing support roles. Subsequently, we present findings from a suite of studies on current nursing practice in hospitals in Kenya that illustrate why addressing the workforce deficit is so critical and the complex space within which HCA might operate. Our conclusions draw on the data presented to ignite debate on policy issues for the development of nursing support roles in Kenya and other LMICs.

## Reference framework: nursing support roles in the UK and EU

### Nurses and HCAs

Many healthcare systems internationally have seen significant changes in the overall clinical human resource base, with task substitution and a profusion of new roles. Nurses are subject to a range of pressures from financial stringency, unmet demand, increasingly technocratic requirements and innovations, and the need to provide care for hospital inpatients with more complex needs. Nurses are increasingly supported by HCAs, or other clinical support workers who are not fully qualified nurses. Whilst this is an international trend across many countries [11], there is considerable variation.

Table 1 provides an overview of the EU position. The countries were selected to be representative of a range of examples in relation to HCAs with varying job titles, training, and regulation [1, 12, 13]. (To aid comparability, for our EU data, we draw heavily on research which adopts standardized definitions of roles based on the International Standard Classification of Occupations.) [14] Accurate figures on the numbers employed in HCA roles are difficult to obtain, as HCAs are often unregistered, largely invisible, and under-researched. The Nursing and Midwifery Council in the UK suggested that there had been a 26% increase over 10 years [15]. Table 1 also demonstrates the variation in nursing ratios. The appropriate ratios of nurses to HCAs for different settings and patient groups have rarely been explored.

**Table 1:** Variation in nursing support roles across the EU

Item	Poland	Spain	UK	Austria	Czech Republic	France	Germany*	Finland	Denmark
Number of nurses per 1000 population [6]	5.16	5.51	7.86	7.99	8.07	10.46	12.85	14.26	16.09
Tiers in the nursing system [1, 13]	Two-tier system 1. Registered Nurse 2. Medical Carer	Two-tier system 1. Registered Nurse 2. Nursing Assistant	Three-tier system (since 2017) 1. Registered Nurses 2. Nurse Associate 3. HCA	Two-tier system 1. Registered Nurse 2. Care Assistant	Two-tier system 1. Registered Nurse 2. Hospital Attendant	Two-tier system 1. Registered Nurse 2. Nurse Assistant	Two-tier system 1. Registered Nurse 2. Healthcare Assistant	Three-tier system 1. Registered Nurse 2. Licensed Practical Nurse 3. Care Assistant	Two-tier system 1. Registered nurse 2. Social/Health Care Assistant
HCA or equivalent classification [1, 12]	Medical Carer (Opiekun medyczny)	Nursing Assistant (técnico en cuidados auxiliares de enfermeria)	Health Care Assistant; Nursing Assistant; Care Assistant; Clinical Support Worker	Care assistant (Pflegehelfer/-in) Home helper in care homes	Hospital Attendant (Ošetřovatel)	Nurse assistant (Aide Soignant/e)	Health care assistant-varies by state e.g. Certifies care Assistant	Care Assistant (Lähihoitaja)	Social/Health Care Assistant (Social- og sundhedsassisten)
Training period for HCA [1, 12]	2 years	8 months approx	No statutory requirement	1600 h	4 years	10 months	2 years approx	Several months; no recognised qualification	8–12 months
Mode of regulation of nursing support workers [1, 13]	National for Medical Carers. Ministry of Health regulates training	National law on duties of care Assistants. Ministry of Health regulates training	National for Nurse Associates trained and regulated by Royal College of Nurses No national regulation for HCAs	Federal law on duties and training of Care Assistants	National and licensed duties and training	National for Nurse Assistants, regulation via Ministry of Health	Federal law regulates training	National and Licensed Practical Nurses No national regulation for Care Assistants	National for Social/Health Care Assistants with compulsory registration and license

*Training regulations may differ by State

### Training and regulation for HCAs

In the EU, the HCA profession is regulated in 14 countries, and HCA education and training is regulated in 22 EU countries. Mandated training varies between 4 years in Slovakia to 2 years in Hungary and 6 months in Italy, and is not a statutory requirement in the UK [1, 12] (Table 1 provides examples). Common elements of training are basic hygiene, infection control, basic nursing skills, interpersonal communication, personal care, storage and cleaning of equipment, and health and safety [12]. Some HCAs will learn human anatomy, physiology, and pathology. There are significant differences in the balance of skill-based to theory-based training.

Regulation means that the knowledge requirements and performance outcomes are set and training is obligatory. The scope of the HCA’s role is specified (i.e., the total number of learning outcomes which they possess) and relates to the country-specific standards. Key regulatory issues are whether the role incumbents should be registered and who should regulate [1, 13]. These questions impinge on professional dynamics and the role of national, professional nursing bodies. Table 1 illustrates varied systems of regulation and these variations in regulation and accountability by country require further in-depth research.

### Task shifting in the UK: the roles and tasks of HCAs

Using UK research on HCAs, we explore the role in practice; bearing in mind in this country, they are not regulated. Table 1 shows the nurse-to-population ratio and job titles. The number of advertised vacancies in ‘nursing and midwifery’ in the NHS is at least 40,000 [6], excluding nurses working in the private sector and care homes.

The roles vary according to the setting. Authors have underlined that in relation to nurses, HCAs performed the most direct care, while nurses performed by far the highest percentage of technical tasks [11]. However, HCAs qualifications are not aligned with the tasks undertaken. HCAs’ core tasks were observations on patients, personal care with feeding, dressing, and mobility, and providing essential emotional support and assisting other clinical professionals. And these tasks were common to the roles of HCAs across the EU [7]. Kessler et al. [11] distinguished five “types” of HCAs which were labelled bed-side technician; ancillary; citizen; all-rounder and expert, underlining the “fuzzy” boundaries of the role. Role differences were accounted for by the range of professionals available on the ward, the extent to which roles overlapped and the shift worked.

Research has displayed that nurses generally valued the HCAs’ contribution to patient care and their help to nurses in performing their roles [11]. Nurses described HCAs as ‘the backbone of the ward’ (p. 745) [16]. HCAs were mostly perceived as taking on routine tasks and being an ‘extra pair of eyes’. Nevertheless, nurses highlighted issues, including the additional burden of supervising HCAs’ work and instances of HCAs overreaching role boundaries.

Despite their critical role in sustaining the system, in the UK, HCAs are not well remunerated [11]. The reasons for this may be partly due to the historical medical hierarchy with HCAs at the lowest level and classed as ‘unskilled’ labour, though some HCAs have relevant National Vocational Qualifications.

### Skill mix

Despite the evidence that the number of nurses and the level of nurse training impact the quality of care and patient outcomes, there has been limited research on the appropriate mix of nurses and HCAs in any given context [7]. Therefore, the growth in the employment of HCAs across the EU has occurred with minimal understanding of the impact on the standards of care. In limited European research, in six EU countries, Belgium, England, Finland, Ireland, Spain, and Switzerland, the average percentage of nurses was 66% and ranged across hospitals from 41 to 87%. Nursing skill mix in NHS hospitals is shown to be one of the lowest in Europe, with variability between 47 and 79% [7], though the Royal College of Nurses recommends a ‘benchmark’ of 65% registered nurses as a proportion of total nursing staff in general hospital wards [17]. The evidence suggests that the design of skill mix to determine the appropriate deployment of HCAs relative to nurses and clinicians in hospitals is now an urgent priority. As many HCAs also work in long-term care and home care, skill-mix policies and deployment norms also need to be adapted to different healthcare sectors and their organizations. The Royal College suggests that nurses should delegate to HCAs who have undergone training and be accountable for that delegation, but no mandated training exists.

This evidence suggests that European countries facing cost constraints and health workforce shortages consider that the employment of HCAs is necessary for sustainability. Despite the widespread employment of HCAs, the skill mix of registered nurses and HCAs applicable to specific patient groups and health care sector is rarely considered or understood. There are further crucial issues in the training, accountability, and systems of regulation for HCAs.

Next, we present research evidence on current nursing practices in Kenya and explore task shifting to minimally trained support workers.

## Nursing practice and the lack of nursing support in Kenya

### Missed care

All of our studies focus on neo-natal wards and underlined that the nurses and midwives worked under conditions of high demand and shifts were frequently under-resourced [2–5]. The nursing ratios in neo-natal wards are typical of other inpatient departments, both adult and children’s wards. Typically, on these units, for between 20 and 40 babies, there would be 3–4 nurses on the morning shift (7.30 am–12.30 pm); 2–3 nurses on the afternoon shift (12.30 pm–6.30 pm), and only 1–2 nurses on the night shift (6.30 pm–7.30 am). (UK nurse-to-patient ratios in similar wards would be between 1:1 and 1:4 depending on patient acuity). Nurses often consciously or unconsciously had to prioritize the care they provided, resulting in some tasks being left undone (missed care). One study identified missed care and the findings indicated on average 40% of nursing care was missed with potentially serious effects on patient safety and outcomes [2]. Examples of frequently missed tasks included nursing review of newborns (38%), cord care (38%), and turning/repositioning (38%). The data illustrated that even the checking of vital signs in acutely ill babies was sometimes missed. The high levels of missed care were strongly related to the low nurse-to-baby ratios observed demonstrating the critical need to expand the nursing workforce.

### Task shifting to untrained helpers

Another study [3] surveyed nurses’ perceptions of the criticality of tasks to explore task shifting. The data suggested that nurses’ most time-consuming tasks were: dealing with emergencies, medical/nursing record documentation, vital signs monitoring, attending ward rounds, and preparing drugs and feeds. Nurses performed time-consuming but relatively low-skilled time-consuming but relatively low-skilled tasks, such as monitoring vital signs and preparing feeds. While no legal provisions for task shifting existed at the time of the survey, informal task shifting to mothers (care providers) and ‘patient attendants’ (support staff, especially in the private sector) was reported by nurses. The range of tasks delegated to mothers included: feeding (naso-gastric tube and cup feeding), weighing of babies, cord care, and feeding chart documentation. These delegated tasks show considerable similarity to tasks delegated to HCAs in the EU [11, 12]. Though involving mothers as care-givers may be perceived as family-centred care which has considerable benefits, evidence from this research suggested that little or no training was provided to mothers in how to deliver these tasks.

Nzinga et al. [4] used qualitative analysis to elucidate the process of task shifting, and nurses’ routines and decision-making processes. To cope with a working environment of intense pressure and to manage the almost “impossible job” of being a neo-natal nurse, nurses developed a form of ‘subconscious triage’ [4]. This was characterized by forming tasks into an implicit hierarchy with technical tasks prioritized over bed-side nursing. The term ‘subconscious triage’ describes the process by which nurses made continuous, responsive decisions on the allocation of a precious resource, their time, and expertise, under extreme pressure. Typically, nurses described how they prioritized the babies who were very sick. It was observed that they often performed aspects of bed-side care while ‘en route’ to technically difficult, clinical tasks. And it is noted that bed-side care is a core task for HCAs in the EU [1, 11]. Instances of delegation and task shifting were observed in all units. Normally, the tasks which were delegated were deemed non-expert and were sanctioned, but not supervised by a senior nurse. Tasks were delegated to nursing students, to mothers and to untrained support staff, such as cleaners.

### Nurses’ coping mechanisms

With an overwhelming workload, nurses adopted collective coping mechanisms and a degree of rationed care [5]. Throughout a substantial body of research, it was observed that nurses coped by working to routines, categorising patients and by covering for each other and themselves in their record keeping. Nurses used documentation as a way of stepping back from the ‘impossibility of it all’ and to mentally ‘escape’ [5]. Collectively, they were very flexible with colleagues over tardiness and absences (sometimes ignoring or ‘looking past’ the fact that colleagues might be working as locums or training unofficially). Nurses remained interested in nursing and career progression. Nurses undertook self-funding of courses, taking locum jobs to supplement their salaries, thus exacerbating problems with tired nurses on duty (especially problematic when nurses sometimes fell asleep on long night shifts). Social relations and empathetic understanding led to informal practices of nurses covering for others during shifts [4, 5]. The nurses exhibited resourcefulness, so ‘one use’ parts would be re-cleaned, machines shared, and domestic heaters drawn into service. A lack of job descriptions led to a blurring of clinical and non-clinical roles with trained nurses collecting medicines or consumables as they believed they were the ‘last line of defence’, since other support and clerical roles had been stripped from the system [5].

The Kenyan evidence displays a health care system under considerable strain with shortages of key human resources including nurses. Necessarily, nurses have to adopt strategies to prioritize their workloads. Nurses perform ‘subconscious triage’ prioritizing technical tasks and care for the sickest babies and delegate tasks to less skilled staff, to ‘available’ others, such as mothers and ancillary staff of all kinds. Task shifting is happening informally and is unregulated. Informal norms of social support were well embedded and unlikely to change.

## In conclusion: will employing health care assistants provide an appropriate solution to nursing workforce deficits in Kenya?

With many countries facing health workforce shortages, especially in nursing, the importance of HCAs in modern health systems is expected to grow. With or without regulation, informal task shifting is occurring in Kenya. As illustrated in the EU data and Table 1, there is considerable variability in the choices made by governments to support nurses across the EU which demonstrates potential routes Kenya or other LMIC might follow. We see examples of HCAs without training; of HCAs employed on tasks which do not match their skills and of units employing higher proportions of HCAs to nurses than may be safe. The potential risks and limited evidence base, especially on the appropriate skill mix, suggest that both developed countries and LMICs should design and prospectively evaluate evidence-based, context-specific HCA workforce policies and assess outcomes as well as potential cost savings.

In particular, we suggest a need for debate for countries like Kenya on:

- Where trained nurses are available, but public-sector recruitment is failing to address the clear gaps in staffing, is it realistic to propose that the only long-term solution is investing much more heavily in employing more nurses? The inexorable trend towards the widespread employment of HCAs in the EU suggests that even better resourced healthcare systems have considered it necessary to sustainability to utilise HCAs in some form to support nurses.
- Missed care clearly impacts outcomes and endangers patients. However, the variability in skill mix in the EU clearly suggests that multiple options might be explored to establish the appropriate skill mix for both general, county hospitals and specialist tertiary units in Kenya.
- HCAs cannot replace nursing staff, but defining their roles will impact the ratios of nurses to HCAs needed, based on the acuity or complexity of patients’ needs. Establishing an evidence base for the skill mix needed in hospitals in Kenya and other LMIC that promotes quality and safety is vital [3, 7].
- If, based on a determination of the unmet need, a formal cadre of HCAs were introduced will such an innovation alone be sufficient to improve care standards? Attention and indeed actions might be needed to address the current social coping mechanisms and organisational norms that have evolved sometimes with potential negative effects as illustrated by research.
- The majority of EU countries have adopted a regulated and trained support cadre of HCAs. Even an unregulated HCA cadre, as in the UK, has been demonstrated to free nurses’ time to focus on critical tasks. However, the UK also displays the negative aspects of a lack of training and regulation with limited matching of skills to tasks. In Kenya, an unqualified, unlicensed, and unregulated version has been emerging in the private sector and task shifting to untrained helpers is occurring in public hospitals. How should Kenya respond to formalising training and regulation, and who should lead this? Is there capacity to take on such responsibilities and how will these impact efforts to further professionalise nursing?
- What might be shorter term activities in Kenya where the role of HCAs might be considered? Local stakeholders might work to see if a scope of practice and relevant training for HCAs might be agreed for the specific context of inpatient county hospital care. Here, the examples from the EU would be of direct use. Policymakers could examine minimum, average, and maximum examples of EU training schemes and make choices. Subsequent steps might include characterizing existing innovations in staffing in the private sector and establishing a register for training schemes and later HCAs who are ‘licensed’ practitioners.
- While focusing on HCAs, we must clearly not forget the high workloads and difficulties facing nurses in Kenya and similar settings. Our research is also a reminder that nurses in Kenya retain their own career aspirations. These aspirations need to be acknowledged and thought given to medium-to-longer term objectives to enhance career development for both nurses and HCAs. If nurses and HCAs do not feel valued, then this may lead to labour turnover.

And we reiterate and highlight broader debates which emerge:

- How to translate skill-mix requirements into an appropriate specification for the optimal healthcare team is a challenging issue, often neglected.
- How can countries build on current evidence to formalise training and regulation, and who should be given responsibility for this?
- The introduction of a new HCA cadre could be limited in scale and evaluated. Evidence suggests that new cadres introduced in Sub-Saharan Africa have rapidly expanded their roles [18]. Stakeholders in the context including nurses and clinicians should join policy makers in a consultation process, so the concerns of all parties are acknowledged and addressed as ‘task shifting’ can be a contested process [5, 12]. Rigorous evaluation could then provide a foundation for specifying long-term training, regulation, and implementation planning.
- The evaluation of HCA implementation needs to take account of the fact that they will enter a multi-professional setting and will have to find their niche, adapting to continuous changes in the health system. This will impact the existing professions whose scope of work may be changing, so developments in regulatory frameworks to enable task shifting need to be flexible and adapt over time.

Building on the available evidence, we argue that an overarching issue is the need to acknowledge that even in HICs, a perfect system—full of the highest trained professionals—may not be affordable. Therefore, context-specific compromises should be sought and rigorously evaluated to strengthen each healthcare system.

## Data Availability

This commentary article is not based on a database.

## Abbreviations

EU: European Union
HCA: Health Care Assistant
HIC: High-Income Country
LMIC: Low- and Middle-Income Countries
WHO: World Health Organization

## Availability of data and materials

This commentary article is not based on a database.
